# The grass pollen season 2015: a proof of concept multi-approach study in three different European cities

**DOI:** 10.1186/s40413-017-0163-2

**Published:** 2017-09-12

**Authors:** Maximilian Kmenta, Katharina Bastl, Uwe Berger, Matthias F. Kramer, Matthew D. Heath, Sanna Pätsi, Anna-Mari Pessi, Annika Saarto, Barbora Werchan, Matthias Werchan, Reinhard Zetter, Karl-Christian Bergmann

**Affiliations:** 1 Department of Oto-Rhino-Laryngology, Medical University of Vienna, Währinger Gürtel 18-20, 1090 Vienna, Austria; 2 Department of Paleontology, University of Vienna, Geozentrum UZA II, Althanstraße 14, 1090 Vienna, Austria; 3 Bencard Allergie GmbH, Leopoldstraße 175, 80804 Munich, Germany; 4 Allergy Therapeutics Ltd, Dominion Way, Worthing, UK; 5 Aerobiology Unit, University of Turku, 20014 Turun yliopisto, Turku, Finland; 6 Foundation German Pollen Information Service, Charitéplatz 1, 10117 Berlin, Germany; 7 Department of Dermatology, Venerology and Allergology, Charité-Universitätsmedizin, Charitéplatz 1, 10117 Berlin, Germany

**Keywords:** Grass pollen allergy, Symptom data, Phenology, Patient’s Hayfever Diary, Pollen exposure chamber

## Abstract

**Background:**

Grasses release the most widespread aeroallergens with considerable sensitization rates, while different species produce several pollen concentration peaks throughout the season. This study analyzed the prevalence of grass species in three different European city areas and compared the flowering period of these species with daily pollen concentrations and the symptom loads of grass pollen allergy sufferers.

**Methods:**

The most prevalent grass species in Vienna (Austria), Berlin (Germany) and Turku (Finland) were studied and examined by use of three different approaches: phenology, pollen monitoring and symptom load evaluation. A mobile pollen exposure chamber was employed to observe reaction patterns of grass pollen allergy sufferers to three common grass species evaluated in this study versus placebo.

**Results:**

Common meadow grass (*Poa pratensis*) and the fescue grass species (*Festuca* spp.) are important contributors within the grass pollen season. The pollination period of orchard grass (*Dactylis glomerata*) and false-oat grass (*Arrhenatherum elatius*) indicated a greater importance in Berlin and Vienna, whereas a broader spectrum of grass species contributed in Turku to the main pollen season. The standardized provocation induced a nasal symptom load, reduction in nasal flow and increased secretion, in contrary to the placebo control group in grass pollen allergic subjects.

**Conclusion:**

The phenological observations, pollen measurements and symptom data evaluation provided unique insights into the contribution of multiple grass species in different European regions. All investigated grass species in the provocation induced rhinitis symptoms of comparable significance, with some degree of variation in symptom patterns.

## Background

Grass pollen allergy is a global problem with sensitization rates up to 30% depending on climate and region [1], [2]. Up to now, eleven groups of grass pollen allergens have been identified including major (> 50% sensitization rate) and minor allergens (< 50% sensitization rate) [1]. In Austria, grass pollen allergy is the most common pollen allergy and more than 50% of all pollen allergy sufferers are sensitized in the eastern part of the country [3]. According to [4], grass pollen is the most important allergen in the adult German population (sample = 7025 participants) with a sensitization prevalence of 18,1%. In Finland, grasses are the second most important cause of pollen allergy after birch and the proportion of Finnish people with clinical symptoms from grass pollen is at least 10–12% [5, 6]. The considerable frequency of grass pollen allergy is owed to the nearly ubiquitous distribution of grasses. Grasses (Poaceae) are one of the largest plant families in the world with more than 10.000 species [7]. Grass dominated habitats cover up to 40% of the earth’s vegetation [8] and extensive cross-reactions among allergens of different grasses are documented [9]. In addition, the different biochemical attributes of component grass species are known [10]. Knowledge in the contribution of single grass species to allergic reactions and sensitization profiles is generally limited. However, one rare study has shown a varying correlation of clinical symptoms to different pollen [11]. It remains unclear if and how those species are responsible for the progress of a pollen allergy under natural conditions. A major problem in the aerobiological routines is the uniform morphology of grass pollen grains [12]. Hence, the identification of different grass species in pollen counting routines is hardly feasible and leads to subjective interpretations in species determination in the optical light microscope [13]. To overcome this problem, several southern European studies combined daily grass pollen concentrations with the approach of phenology: the visual observation of plant life cycles (including flowering conditions) in the field [14] and identification of the grass species that contribute the most to daily pollen concentrations [15–18]. A recent pilot study evaluated, for the first time, the profile of a single grass pollen season in a central European city, by combining daily grass pollen concentrations, grass species observations in the field and crowd sourced symptom data from the Patient’s Hayfever Diary [19]. The same approach was used in the study presented herein, comparing the progress of the grass pollen season in 2015 across three different European cities and thus to test its applicability in different regions: Vienna (Austria), Berlin (Germany) and Turku (Finland). All locations are situated in the terrestrial “temperate broadleaf and mixed forest” biome according to the World Wildlife Fund’s (WWF) classification of vegetation [20]. Berlin is the largest city in this study with more than 3.5 Million inhabitants built on several small plateaus in the transition of the maritime to the continental climate (Cfb climate in [21]). Vienna is situated along the Eastside of the Alps passing into the Pannonian plain in the transition of the oceanic to the continental climate (Cfb climate in [21]) and records more than 1.8 Million inhabitants. Turku is the smallest city in this setting with less than 200.000 inhabitants, situated close to the Baltic Sea and in the influence of a cold-temperate climate (Dfb climate in [21]). Moreover, the three cities were chosen to evaluate the methodological approach in different settings and to assess the influence of biogeographical parameters. In addition to grass species observations in the field, crowd-sourced symptom data and daily pollen concentrations - a novel feature was introduced: three different grass species that were found to be highly relevant in their contribution to the grass seasons in this study (*Dactylis glomerata*, *Festuca pratensis*, *Phleum pratense*) were used in a mobile pollen exposure chamber to observe whether pollen allergy sufferers exhibit different reactivity profiles to pollen from each grass species.

## Methods

### Phenology

Phenological observations and identification of different grass species were performed once to twice a week in different locations of Vienna, Berlin and Turku. Different urban habitats in extensive observation sites were chosen to cover a representative range of grass species. In Vienna the location of “Steinhofgründe” (more natural) and the location of “Neue Donau/Wasserpark” (more urbanized) were selected since both locations performed well in the recently conducted pilot study [19]. Moreover, a third location next to the pollen monitoring station in the phenological garden of the Vienna meteorological service was included. The total area of all observation sites in Vienna was more than 80.000m^2^. In Berlin four observation sites for phenological observations were chosen: “Nordbahnhof” (urban wilderness park), “Tempelhof Projekt” (city park), “Botanischer Garten” (meadows of the Botanical Garden Berlin-Dahlem) and “TU Berlin” (phenological garden). The total area of all observation sites in Berlin amounts to approximately 7000m^2^. In Turku three observation sites have been selected: “University area” (city center), “Skanssi” (5 km distance to the city center) and “Tuorla” (agricultural zone 15 km apart from Turku) with a total area of 60.000m^2^. All observation areas are located in the city borders of the respective cities and are in close distance to the pollen monitoring stations. In Vienna and Berlin random fields with a surface of approximately 4 m^2^ were studied per location for the field observations. However, it has to be mentioned that the location of the random fields could be set wider apart due to governmental lawn mowing activities (see limitations). In Turku the observation areas were defined on an urban to agricultural gradient and inside each study area the observed sites were selected randomly (one site in University area and Skanssi, three sites in Tuorla). Five different phenological phases have been defined to determine the pollination periods of each grass species (detailed definition in [19]). These phases were translated into international BBCH phenological phases [22]. Only more than 25 individuals per grass species and defined area were examined to evade observing poorly distributed grasses at the respective surface [16].

### Pollen measurements

Daily pollen concentrations were assessed with volumetric pollen and spore traps of the Hirst design [23] in the three European cities. The collected data were evaluated according to the minimum recommendations of the European Aeroallergen Society [24] to ensure high data quality. The main pollination period of the grass pollen season 2015 was defined by applying the standardized season definition of the European Aeroallergen Network (EAN). Therefore, the season starts at the day with 1% of the cumulative annual total grass pollen count and ends at the day of 95% of the total annual pollen count.

### Crowd sourced symptom data

Crowd sourced symptom data in all cities was used from the Patient’s Hayfever Diary (https://www.pollendiary.com). This pollen diary is a free web-based online diary and records symptoms of users suffering from pollen allergies. Data from this diary is highly practicable and was used in several scientific studies [25–28]. At the moment the Patient’s Hayfever Diary is available in thirteen European countries. Users fill in a validated questionnaire and indicate the symptom severity of eyes, nose and lungs including medication use. A total symptom score can be calculated with this basic information. For the purpose of this study, the data of all users from Berlin, Vienna and Turku exhibiting a positive background correlation to grasses during the grass pollen season in 2015 were included following the methodology of [19]. Hence, only users with a minimum of 10 entries during the main grass pollen season were included into the evaluation. Furthermore, users with a positive background correlation to birch pollen were excluded in Berlin and Vienna, thus to avoid unrealistic symptom loads in the beginning of the grass pollen season due to poly-sensitization and a short overlap of the birch and grass pollen season. In Turku, the grass pollen season and the birch pollen season were not overlapping; hence users were only filtered for a minimum of 10 entries and a positive background correlation to grass pollen. Daily symptom load indices were calculated as described in [25] after user filtering. Thus, the total symptom scores (including all organs, specific symptoms and medication use) of all user entries were normalized to attain daily mean values between 0 and 10 for the respective time period of the grass pollen season.

### Pollen exposure chamber and exposure tests

Exposure tests with pollen from three different grass species (*Phleum pratense*, *Festuca pratensis* and *Dactylis glomerata*; Pharmallerga, Czech Republic) were performed in the mobile GA2LEN chamber in Berlin, which is validated according to current needs and requirements [29]. Eight non-smoking adults (6 female, 2 male; mean age 29 years) suffering from allergic rhinoconjunctivitis since more than two years (pos. Skin prick test of 3 mm or greater and/or an ImmunoCAP score of 2 or greater, and FEV1 of 70% of predicted value or greater, typical hay-fever symptoms during grass pollen season) were exposed double-blinded 4 times (placebo run and pollen from 3 grass species, 4000 pollen/m^3^) on 4 different days with at least 7 days interval for 120 min outside the pollen season. Patients recorded eye and nose (itching, sneezing, running and blocked) symptoms. For every symptom, a score of 0 to 3 was applied (none, mild, moderate, and severe). The total nasal symptom score (TNSS) is the sum of the 4 nasal symptoms (minimum = 0 and maximum = 12). Subjective parameters were recoded at time zero (0) and every 30 min during exposure.

### Statistical analysis

**Fig. 1:**
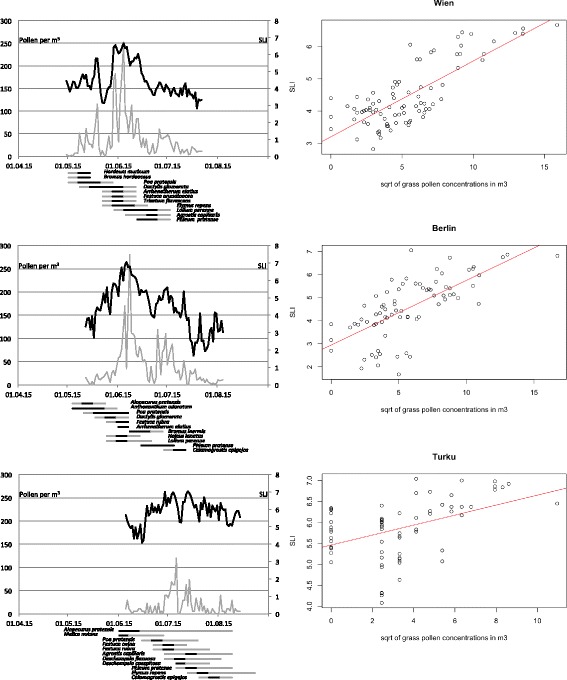
Comparison of daily grass pollen concentrations per m^3^ (left y-axis; *gray line*), daily averaged SLI values from 0 to 7 (right y-axis; *black line*) and the pollination periods of the different grass species including phenological stages 1–3 (1 and 3 *gray horizontal lines*; 2 *black horizontal lines*) during the main grass pollen season 2015 in Vienna (*top left*), Berlin (*center left*) and Turku (*bottom right*). Moreover, graphs of the linear correlation model of daily SLI observations (y-axis) and the square root of daily pollen concentrations per m^3^ (x-axis) for Vienna (*top right*), Berlin (*center right*) and Turku (*bottom right*)

The software R in version 3.3.1 was used for statistical analysis. For the three locations a linear regression model was estimated for the symptom data with the square root of pollen concentration data as only independent variable (Fig. 1). The residuals of these linear regression models were used in an analysis of variance (ANOVA) with the pollination periods of the most prevalent grass species in every location to assess the impact of single grass species on the symptom data. Moreover, an additional analysis of variance (ANOVA) was applied on the linear model of square root of pollen concentration data against the pollination periods of the prevalent grass species to examine the impact of single grass species on the grass pollen concentrations during the grass pollen season.

## Results

### Field observations in the three European cities

In total, 34 grass species could be identified and observed in Vienna, Berlin and Turku (Table 1). Eleven species were present in all European cities. For reasons of clarity and comprehensibility only these 11 species that exhibited the highest distribution at the observation sites in each location are discussed herein. The flowering of meadow grass (*Poa pratensis*) occurred at the beginning of the main grass pollen season and the group of fescue grasses (*Festuca* spp.) flowered during the peak of all grass pollen seasons concerned. Detailed information of all grass species observed at the respective locations including vegetation cycle, start and end of the pollination period and distribution at the observation sites is presented in Table 1. Only the phenological phases where pollination is likely (phase 1–3; see [19]) have been included in Fig. 1.

**Table 1:** Observed grass species in the phenological areas of Vienna, Berlin and Turku including start and end of the pollination period as well as the species life cycle (A = annual, P = perennial) and the distribution at the local observation sites (number of appearance/maximum number of observation sites)

Observed grass species	Cycle	Start of the pollination period	End of the pollination period	Distribution (appearance at phenological sites)	Concerned cities
*Agrostis capillaris*	P	week 24	week 27	2/3	Vienna
		week 25	week 27	2/4	Berlin
		week 27	week 32	3/3	Turku
*Alopecurus pratensis*	P	week 17	week 20	1/3	Vienna
		week 19	week 21	2/4	Berlin
		week 23	week 32	3/3	Turku
*Antoxanthum odoratum*	P	week 17	week 19	1/3	Vienna
		week 19	week 22	2/4	Berlin
		week 23	week 25	2/3	Turku
*Arrhenatherum elatius*	P	week 22	week 24	3/3	Vienna
		week 23	week 23	3/4	Berlin
		week 27	week 29	2/3	Turku
*Brachypodium sylvaticum*	P	week 26	week 28	1/3	Vienna
*Briza media*	P	week 22	week 23	1/3	Vienna
*Bromus erectus*	P	week 22	week 24	1/3	Vienna
*Bromus hordeaceus*	A	week 19	week 20	2/3	Vienna
		week 20	week 22	3/4	Berlin
*Bromus inermis*	P	week 24	week 26	2/4	Berlin
*Bromus sterilis*	A	week 20	Week 22	2/4	Berlin
*Calamagrostis arundinacea*	P	week 29	week 33	2/3	Turku
*Calamagrostis epigejos*	P	week 23	week 26	1/3	Vienna
		week 27	week 28	2/4	Berlin
		week 30	week 32	3/3	Turku
*Cynodon dactylon*	P	week 29	week 33	1/3	Vienna
		week 28	week 35	1/4	Berlin
*Cynosurus cristatus*	P	week 25	week 26	1/3	Vienna
*Dactylis glomerata*	P	week 20	week 24	3/3	Vienna
		week 21	week 23	4/4	Berlin
		week 27	week 32	2/3	Turku
*Deschampsia cespitosa*	P	week 28	week 30	3/3	Turku
*Deschapsia flexuosa*	P	week 27	week 31	3/3	Turku
*Elymus repens*	P	week 22	week 25	2/3	Vienna
		week 29	week 34	3/3	Turku
*Festuca arundinacea*	P	week 22	week 24	3/3	Vienna
		week 23	week 24	1/4	Berlin
*Festuca ovina*	P	week 26	week 28	1/3	Turku
*Festuca pratensis*	P	week 22	week 24	1/3	Vienna
		week 26	week 28	2/3	Turku
*Festuca rubra*	P	week 22	week 23	4/4	Berlin
		week 26	week 30	3/3	Turku
*Helictotrichon pubescens*	P	week 20	week 21	1/3	Vienna
*Holcus lanatus*	P	week 23	week 24	1/3	Vienna
		week 22	week 24	2/4	Berlin
*Hordeum murinum*	A	week 19	week 20	2/3	Vienna
		week 20	week 23	1/4	Berlin
*Lolium perenne*	P	week 23	week 27	3/3	Vienna
		week 22	week 25	2/4	Berlin
		week 29	week 33	3/3	Turku
*Melica nutans*	P	week 23	week 26	1/3	Turku
*Phalaris arundinacea*	P	week 29	week 32	2/3	Turku
*Phleum pratense*	P	week 25	week 27	2/3	Vienna
		week 25	week 27	2/4	Berlin
		week 28	week 32	3/3	Turku
*Phragmites australis*	P	week 33	week 36	2/3	Turku
*Poa annua*	A	week 23	week 34	3/3	Turku
*Poa nemoralis*	P	week 27	week 29	1/3	Turku
*Poa pratensis*	P	week 19	week 22	3/3	Vienna
		week 20	week 23	3/4	Berlin
		week 25	week 29	3/3	Turku
*Trisetum flavescens*	P	week 22	week 24	2/3	Vienna
		week 23	week 23	1/4	Berlin

#### Vienna

In Vienna, phenological observations began at the end of April (week 17) and lasted until mid-August (week 33). In total, 22 grass species were observed at all observation areas of Vienna (Table 1). At the start of May, false barely grass (*Hordeum murinum*), false brome grass (*Bromus hordeaceus*) and common meadow grass (*Poa pratensis*) began flowering and initiated the main grass pollen season (Fig. 1). By mid-May, orchard grass (*Dactylis glomerata*) started flowering in concert with the first pollen peaks, followed by the flowering of false-oat grass (*Arrhenatherum elatius*), tall fescue grass (*Festuca arundinacea*), yellow oat grass (*Trisetum flavescens*) and couch grass (*Elymus repens*) at the end of the month (Fig. 1). Perennial rye-grass (*Lolium perenne*) flowered later and produced, together with orchard grass, the longest pollination period of all species observed ranging from the end of May until the beginning of July (Fig. 1). Common bent grass (*Agrostis capillaris*) and Timothy grass (*Phleum pratense*) began their flowering period in mid/end of June and thus were the relevant contributors during final phases of the grass pollen season (Fig. 1). Only *Poa pratensis*, *Dactylis glomerata*, *Arrhenatherum elatius*, *Festuca arundinacea* and *Lolium perenne* were highly prevalent at all observation areas included.

#### Berlin

In Berlin, phenological observations began at the end of April (week 17) and lasted until the end of August (week 35). In total, 18 grass species were observed at all observation areas of Berlin (Table 1). At the beginning of May, sweet vernal grass (*Anthoxanthum odoratum*) and meadow foxtail grass (*Alopecurus pratensis*) started flowering (Fig. 1). By mid-May, the main grass pollen season was initiated by the flowering of orchard grass (*Dactylis glomerata*) and the full flower of common meadow grass (*Poa pratensis*) (Fig. 1). The first and main peak of the grass pollen season was introduced in the beginning of June by a variety of grass species including the latter two, together with red fescue grass (*Festuca rubra*), false-oat grass (*Arrhenatherum elatius*), Yorkshire fog (*Holcus lanatus*), perennial rye grass (*Lolium perenne*) and smooth brome grass (*Bromus inermis*) (Fig. 1). The second and lower peak in the season by the end of June/beginning of July was accompanied by the flowering of Timothy grass (*Phleum pratense*) and wood small-reed grass (*Calamagrostis epigejos*) (Fig. 1). Only *Dactylis glomerata* and *Festuca rubra* were highly prevalent at all observation areas.

#### Turku

In Turku, phenological observations began mid-May (week 21) and lasted until the beginning of September (week 36). In total, 21 grass species were observed at all observation areas (Table 1). The first grass species that started flowering in the turn of May and June were meadow foxtail grass (*Alopecurus pratensis*) and mountain melic grass (*Melica nutans*) (Fig. 1). The main grass pollen season and the subsequent first peak in mid-June was caused by the main flowering of common meadow grass (*Poa pratensis*), sheeps fescue grass (*Festuca ovina*), red fescue grass (*Festuca rubra*), tufted hair grass (*Deschamspia flexuosa*) and wavy hair grass (*Deschampsia caespitosa*) (Fig. 1). The second pollen peak in mid-July and the grass flowering in the latter half of July were contributed by common bent grass (*Agrostis capillaris*), Timothy grass (*Phleum pratense*) and couch grass (*Elymus repens*) (Fig. 1). The grass pollen season faded into August and only wood small-reed grass (*Calamagrostis epigejos*) had its flowering peak during the last week of July and the first week of August (Fig. 1). No more pollen peaks were observed after July. Only *Alopecurus pratensis* and *Phleum pratense* were highly prevalent at all observation areas and sites included.

### Daily pollen concentrations in the three European cities

Pollen concentration measurements were taken continuously at all pollen monitoring sites (Vienna, Berlin, Turku) during the grass pollen season of 2015, resulting in a complete record of grass pollen concentrations. The characteristics of the three grass pollen seasons are described as follows, since differences could be observed concerning the pollen levels (season intensity) and the pollen curve (season progress). Only minor concentrations occurred in the period before and after the main grass pollen season (data not shown).

The main grass pollen season started on the 30th of April 2015 and ended on 22nd of July 2015 in Vienna. The peak day was the 4th of June, with a concentration of 253 grass pollen per m^3^. In total, 7 days with a concentration exceeding 100 grass pollen per m^3^ air were recorded. Indeed, this concurs with the main spiking in pollen peaks that were observed during the season from the end of May and through June, as a result of the flowering in a number of the grass species described in section 3.1.1. The grass pollen season of 2015 in Vienna was an intense season (above average) in comparison to the last five years.

The main grass pollen season started on the 12th of May 2015 and ended on 4th of August 2015 in Berlin. The peak day was the 6th of June 2015 with a concentration of 280 grass pollen per m^3^. In total, 9 days with a concentration exceeding 100 grass pollen per m^3^ air were recorded. Two main peaks could be observed during the season at the beginning/mid of June and end of June/beginning of July, attributed to a variety in flowering of grass species during this time (see Berlin section). The grass pollen season of 2015 in Berlin was an intense season (above average) in comparison to the last five years.

The main grass pollen season started on the 5th of June 2015 and ended on 14th of August 2015 in Turku. The peak day was the 6th of July with a concentration of 120 grass pollen per m^3^. In total, only one day (the peak day) with a concentration exceeding 100 grass pollen per m^3^ air was recorded. Two main peaks could be observed during the season in the end of June/beginning of July and mid of July attributed to a variety in flowering of grass species during this time (see Turku section). The grass pollen season 2015 in Turku was an average season in comparison to the last five years in terms of peak value and total amount of pollen, however the number of days with zero grass pollen in the air was somewhat higher.

### Daily symptom load data in the three European cities

The number of users, after a filtering process, whose symptom data were calculated was 254 (Vienna), 46 (Berlin) and 16 (Turku). The main grass pollen season started in Vienna with a Symptom Load Index (SLI) above 4 and increased with the pollen concentration to the highest recorded value of 6.7, then subsequently decreases to a value of 3 at the end of the main season (Fig. 1). The SLI started in Berlin with a value above 3, peaked with a value of 7 (two days before the grass pollen peak) and decreased to a value of 3 at the end of the main season (Fig. 1). In Turku, a SLI of 5.5 was recorded at the beginning of the season, exceeded by three peaks of ~7 (two during the first and one during the second grass pollen peak) with a subsequent decrease to 5.5 at the end of the main grass pollen season (Fig. 1). It should be noted that grass pollen concentration peaks were well reflected in the pattern of the SLI and that the SLI was similarly high in Turku, despite the lower grass pollen concentrations (compared to Vienna and Berlin).

### Statistical outcome

In all observation sites symptom data and pollen data showed significant correlations in a linear regression model (Fig. 1). The estimated R-square values are 0.6 (Vienna), 0.5 (Berlin) and 0.2 (Turku). Moreover, the *p*-values of <2*10^−16^*** (Vienna), 5*10^−12^*** (Berlin) and 0.00013*** (Turku) elucidates this significant correlation. The analysis of variance presented highly significant *p*-values for several grass species compared to symptom data, pollen data or both. In Vienna, *Arrhenatherum elatius*, *Dactylis glomerata*, *Elymus repens*, *Festuca arundinacea* and *Trisetum flavescens* exhibited highly significant *p*-values suggesting an additional impact on the symptom data and the grass pollen data (Table 2). *Lolium perenne* contributed significantly to the pollen concentration data and *Phleum pratense* demonstrated significant *p*-values compared to the symptom data only (Table 2). In Berlin, *Anthoxantum odoratum*, *Arrhenatherum elatius*, *Festuca rubra*, *Holcus lanatus*, *Lolium perenne* and *Poa pratensis* exhibited highly significant *p*-values in symptom and pollen data (Table 2). The *p*-values of *Dactylis glomerata* are highly significant in comparison with the symptom data and *Alopecurus pratensis* and *Bromus inermis* contributed with highly significant values with the grass pollen concentrations (Table 2). In Turku, *Agrostis capillaris*, *Deschampsia flexuosa*, *Festuca rubra*, *Melica nutans* and *Poa pratensis* showed highly significant *p*-values in symptom and pollen data (Table 2). The highly significant *p*-values of *Alopecurus pratensis*, *Deschampsia caespitosa* and *Phleum pratense* suggested an additional influence on the symptom data for these grasses, as well as *Festuca ovina* on the pollen concentrations. Additional statistical information as well as significance rates for all grasses with high prevalence in all cities is displayed in Table 2.

**Table 2:** *P*-values of the analysis of variance from the resiudals of the linear correlation model with the most prevalent grass species at all three cities as well as *p*-values from the second analysis of variance regarding the daily pollen concentrations

Most prevalent grass species	*P*-values ANOVA linear regression model residuals	*P*-values ANOVA square root of pollen concentrations	Occurrence at
*Agrostis capillaris*	0.1479	0.5478	Vienna
	0.001528 **	0.0222 *	Turku
*Alopecurus pratensis*	0.3941	0.001284 **	Berlin
	2.539*10^−06^ ***	0.4861	Turku
*Anthoxantum odoratum*	0.003809 **	0.0008423 ***	Berlin
*Arrhenatherum elatius*	0.0001877 ***	1.182*10^−10^ ***	Vienna
	0.02242 *	0.0007735 ***	Berlin
*Bromus hordeaceus*	0.3722	0.2972	Vienna
*Bromus inermis*	0.3794	0.004911 **	Berlin
*Calamagrostis epigejos*	0.7875	0.07981.	Berlin
	0.4429	0.1188	Turku
*Dactylis glomerata*	0.005651 **	3.456*10^−06^ ***	Vienna
	2.733*10^−05^ ***	0.3172	Berlin
*Deschampsia caespitosa*	0.02357 *	0.2024	Turku
*Deschampsia flexuosa*	6.198*10^−05^ ***	0.011 *	Turku
*Elymus repens*	1.66*10^−05^ ***	2.029*10^−08^ ***	Vienna
	0.1014	0.385	Turku
*Festuca arundinacea*	0.0001877 ***	1.182*10^−10^ ***	Vienna
*Festuca ovina*	0.05804 .	4.741*10^−05^ ***	Turku
*Festuca rubra*	0.0002582 ***	0.002926 **	Berlin
	6.268*10^−05^ ***	9.737*10^−06^***	Turku
*Holcus lanatus*	0.01636 *	1.188*10^−07^ ***	Berlin
*Hordeum murinum*	0.3722	0.2972	Vienna
*Lolium perenne*	0.6543	0.000342 ***	Vienna
	0.04644 *	1.484*10^−07^ ***	Berlin
*Melica nutans*	0.001806 **	0.04513 *	Turku
*Phleum pratense*	0.007979 **	0.6982	Vienna
	0.7379	0.1035	Berlin
	0.03159 *	0.3066	Turku
*Poa pratensis*	0.3846	0.7961	Vienna
	3.217*10^−05^ ***	0.007992 **	Berlin
	0.03531 *	0.001066 **	Turku
*Trisetum flavescens*	0.0001877 ***	1.182*10^−10^ ***	Vienna

Significance codes: 0 ‘***’ 0.001 ‘**’ 0.01 ‘*’ 0.05 ‘.’ 0.1 ‘1’

### Pollen exposure chamber results

**Fig. 2:**
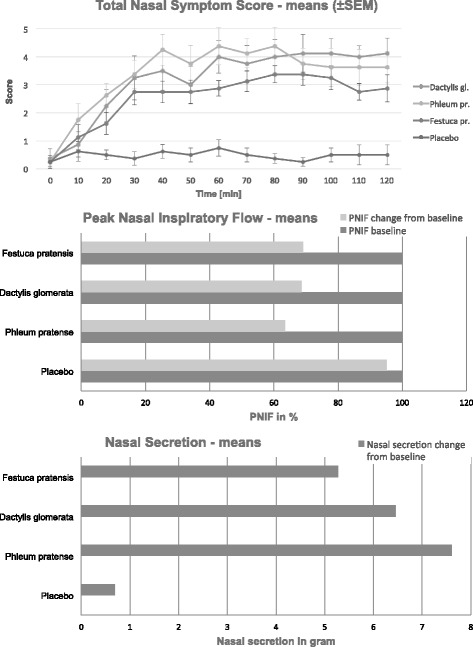
Total nasal symptom scores (mean and SEM) induced by placebo and three different pollen species (*top*), mean values of the reduction of nasal flow by placebo and three different grass pollen species (*center*). And mean values of the reduction of nasal secretion by placebo and three different grass pollen species (*bottom*)

The placebo run induced a TNSS of 0,5 ppt. The TNSS with pollen of *Dactylis glomerata*, *Festuca pratensis* and *Phleum pratense* ranged from 3 to 4,5 points forming a plateau after about 60 min (Fig. 2). A reduction of nasal flow and induction of nasal secretion was induced by pollen, but not in the placebo run (Fig. 2). All grass allergic patients reacted when exposed to different grass species in the EEC. The pattern of symptomatic reaction exhibited some degree of variation between the three grass species, however, in the statistical analyses there is only a significant difference comparing the placebo group versus all of the different grass species, considering a 5% confidence interval in the TNSS (*p*-value for each grass tested vs. placebo <0.00001).

## Discussion

### Interpretation and conclusions

The multi-approach design of this study performed in three different cities provided results concerning grass pollen concentrations, grass flowering periods, grass species’ distribution and their relationship to symptom data. The grass pollen season of 2015 was more intensive compared to a typical season in Austria and Berlin, whereas in Turku the grass pollen season was average. The application in different European regions allowed for the first time insights in the spatio-temporal variation of the grass pollen season and its main contributors. The results from the pilot study in Vienna, 2014 [19] were reproduced in relation to the five main contributors to the pollen season from the phenological point of view (*Poa pratensis*, *Dactylis glomerata*, *Arrhenatherum elatius*, *Fetuca arundinacea* and *Lolium perenne*), as well as the impact on symptom data (*Dactylis*, *Arrhenatherum* and *Festuca* show significant *p*-values on the symptom data). However, differences between the grass pollen season 2014 and 2015 can be noted: *Poa pratensis* did not show significant *p*-values on the symptom data in contrast to previous findings [19]. The reason for this could be the intensive grass pollen season of 2015 which resulted in shorter pollination periods for most of the grass species evaluated (Fig. 1), resulting in high collinearity for a plethora of grass species (Table 2). The grass species composition of Berlin show similarities compared to the species composition of Vienna. In particular, the significant *p*-values of the grass species contributing to the pollen and symptom peaks for *Poa pratensis*, *Arrhenatherum elatius*, *Dactylis glomerata*, *Festuca rubra* and *Lolium perenne* stand out and are comparable to the results of the Vienna pilot study [19]. The daily grass pollen concentrations and the intensity of the symptoms are also comparable (Fig. 1). However, the composition of the most prevalent grass species show variations compared to the results in Vienna (Fig. 1, Table 2). *Alopecurus pratensis*, *Anthoxanthum odoratum* and *Holcus lanatus* showed higher contributions to the pollen concentrations and symptom load data in Berlin compared to results in Vienna (Fig. 1, Table 2). Moreover, *Festuca rubra* is the dominant *Festuca* species (not *F. arundinacea*) and *Bromus inermis* is an additional contributor to the pollen concentrations in Berlin (Fig. 1, Table 2). In Turku the situation was significantly different. The pollen season was less intense with longer pollination periods observed (Fig. 1). Of particular note were the daily grass pollen concentrations that were notably lower even though the intensity of the SLI was comparable to levels documented in Vienna and Berlin (Fig. 1). Marked differences in the composition of grass species, especially regarding the most prevalent species (*Melica nutans*, *Festuca ovina*, *Deschampsia caespitosa*, *Deschampsia flexuosa*) is clearly different from the observation sites of Vienna and Berlin. The statistical analysis demonstrated a significant correlation between daily pollen concentrations and daily SLI data for every European city (Fig. 1; Table 2). Also, in former studies comparable correlations were confirmed [19, 30, 31]. The local vegetation, including the distribution of grass species, is different and therefore it was important to include phenological observations in order to confirm the most important contributors to the grass pollen season in a given location. Moreover, the pollination periods of the most prevalent grass species evidently vary from season to season depending on meteorological parameters, grass species distribution, biogeographical region and climate zones. In addition, the results reveal the variable spatio-temporal distribution and contribution of grass species (Vienna 2014/2015, Berlin 2015 and Turku 2015). The statistical analysis infers that a minimum amount of symptom data is required to more accurately translate meaningful analysis. For example, in the case of Turku, the number of participants in the symptom diary is too low to produce convincing results relating to the normal distribution of residuals in the linear regression model. Hence, although an analysis of variance produced adequate results the *p*-value of the Shapiro Wilks test is insignificant to confirm the results of the ANOVA tests performed. Thus, user numbers should amount to more than 50, or better still, 100 users after the filtering process in order to produce statistically convincing results in unusual pollen seasons. The mobile exposure chamber was included in such a phenology driven study for the first time. The exposure tests documented that pollen from the three tested grass species were able to induce significant nasal symptoms, a reduction in the nasal flow and induction of nasal secretion. It appears that individual grass species induce a degree of variability in patterns of symptoms in grass allergic patients. However, there were no statistical differences in the answer of the subjects between the three pollen species, demonstrating that all three species can induce typical hay-fever symptoms during the grass pollen season that was significant versus placebo.

### Limitations

Green (grassy) areas in metropolis like Vienna or Berlin but also in smaller cities like Turku are restricted and subject to many changes such as construction work, governmental mowing activities, management and economic influences (sale, private properties). These activities may have an effect on field observations due to the loss of available areas resulting in adaptations to local circumstances. In Vienna and Berlin, large areas for phenological observations were captured to enable flexibility in movement within the region (random fields), thus offering a more accurate representation for the grass flora of the respective city [32, 33]. In Turku, only minor parts of grassy areas were affected and hence human activities may not have as pronounced impact on phenological changes. The pollen data obtained is adhering to the minimum recommendations for the evaluation of pollen concentration data [24]. However, aerobiological measurements underlie certain limits and represent only single point measurements. Pollen traps included in this study are positioned on a rooftop - a location ideal to assess pollen concentrations for a region [19]. However, since the pollen concentrations were assessed at a considerable height and not on a level relevant for pollen allergy sufferers, there is much discourse in the literature as to how pollen concentrations differ on rooftop and nose level, thus the indications for a difference should not be neglected ([34–36], pers. observation). The data of the Patient’s Hayfever Diary is crowd-sourced data (easy and fast access, validity through high usage, but minimal information on subject profiles). A filtering process including only significantly positive correlating users to grass pollen without correlation to birch pollen and more than ten entries ensured the quality of the dataset. Nonetheless, not all users entered symptom data over the whole study period. A varying allergic burden of the users, during any pollination period, was previously observed for different aeroallergens [28] and could also be part of an explanation for this, but is difficult to correlate. Users and their medical history remain unknown for the study, as the Patient’s Hayfever Diary conforms to data protection law. While the data for Vienna and Berlin seems numerous enough for a stable fundament, the user numbers in Turku remained too low (significantly lower than in Vienna and Berlin) to state with certainty the result will not change with more data and higher user numbers. Limitations of this study comprise (1) changes in the season of green areas (phenological sites), (2) restrictions of pollen data acquainted by the standard procedures [24] and (3) the nature and number of crowd sourced data.

## Conclusion

A combination of methods such as pollen monitoring, assessment of the flowering period of different grass species, evaluation of symptom data and environmental challenge of patients with pollen from different grass species proved to be useful to estimate the impact of certain grass species during the grass pollen season. This novel approach was applied to three different European cities considering their size (metropolises (Vienna, Berlin) vs. city (Turku)), climate (Cfb vs. Dfb) and grass species composition. This study provides evidence of (1) the necessity of phenology to assess the contribution of different grass species during the grass pollen season, (2) the different symptom levels during the flower of grasses and in different localities, (3) a combination of different methods is necessary to begin to understand the complex connections relevant for grass pollen allergy sufferers and (4) to give adequate advice to both, patients and allergologists.

## Abbreviations

EAN: European aeroallergen network
SLI: Symptom load index
TNSS: Total nasal symptom score
